# Arginine Vasopressin Injected into the Dorsal Motor Nucleus of the Vagus Inhibits Gastric Motility in Rats

**DOI:** 10.1155/2016/4618672

**Published:** 2015-12-30

**Authors:** Jianping Zhu, Lanlan Chang, Jinlu Xie, Hongbin Ai

**Affiliations:** College of Life Sciences, Shandong Normal University, Jinan 250014, China

## Abstract

*Background*. Until now, the effect of arginine vasopressin (AVP) in the DMV on gastric motility and the possible modulating pathway between the DMV and the gastrointestinal system remain poorly understood.* Objectives*. We aimed to explore the role of AVP in the DMV in regulating gastric motility and the possible central and peripheral pathways.* Material and Methods*. Firstly, we microinjected different doses of AVP into the DMV and investigated its effects on gastric motility in rats. Then, the possible central and peripheral pathways that regulate gastric motility were also discussed by microinjecting* SR49059* (a specific AVP receptor antagonist) into the DMV and intravenous injection of hexamethonium (a specific neuronal nicotinic cholinergic receptor antagonist) before AVP microinjection.* Results*. Following microinjection of AVP (180 pmol and 18 pmol) into the DMV, the gastric motility (including total amplitude, total duration, and motility index of gastric contraction) was significantly inhibited (*P* < 0.05). Moreover, the inhibitory effect of AVP (180 pmol) on gastric motility could be blocked completely by both* SR49059* (320 pmol) and hexamethonium (8 *μ*mol).* Conclusions*. It is concluded that AVP inhibits the gastric motility by acting on the specific AVP receptor in the DMV, with the potential involvement of the parasympathetic preganglionic cholinergic fibers.

## 1. Introduction

Arginine vasopressin (AVP) is synthesized by the vasopressinergic neurons of paraventricular hypothalamic nucleus (PVN) and supraoptic nucleus (SON) and plays important roles in water conservation, and so on. Recent studies have demonstrated that AVP is synthesized throughout the brain and can function as a neurotransmitter/neuromodulator and modulate other complicated and varying autonomic function, not just antidiuresis and vascular tone [1–5]. The dorsal motor nucleus of the vagus (DMV) primarily serves parasympathetic functions and consists largely of vagal preganglionic neurons that project to the stomach and control gastric motility [6]. Numerous anatomical and electrophysiological studies have provided evidence for direct and monosynaptic projections from the PVN to the DMV [7–9], which indicates that afferent information from the PVN to the DMV might participate in the process of modulating activities of neurons in the DMV.

In our previous studies, we had noted that water-immersion restraint-related stress in rats activated both vasopressinergic and oxytocinergic neurons in the PVN and caused serious gastric dysfunction. Meanwhile there were AVP and oxytocin (OT) receptors largely expressed in the soma and/or dendrite membranes of the activated neurons in the DMV [10, 11], suggesting the possible involvement of vasopressinergic/oxytocinergic projections from the PVN to the DMV in modulating the gastric functions.

The long-descending oxytocinergic projection from the PVN to the DMV has been reported to serve as a link in the main neural control of the gastric function, indicating the important role of OT in autonomic function. The gastric-related neurons in the DMV have been shown to belong to OT-sensitive neurons [12], which is involved in regulating gastric motility [1, 13]. AVP and OT are regarded as highly related nonapeptides hormones in structure and could combine with their receptors reciprocally [14], indicating that AVP might also be a central regulator of gastric function. However, none has been done so far to investigate the relationship between AVP in the DMV and gastric function, and whether changes in DMV neuron activity initiated by AVP produce a modulation of gastric motility is still not well defined yet. Thus, the present study aimed to investigate whether AVP in the DMV plays a role in regulating the gastric motility. Additionally, the central actions of AVP are mainly mediated by V1 receptors [15], and most of the vagal preganglionic neurons controlling gastric function in the DMV are cholinergic neurons in nature [16]. Therefore, the possible central and peripheral mechanism of AVP on gastric motility were also explored by using AVP receptor blocker (*SR49059*) and preganglionic antagonist (Hexamethonium).

## 2. Materials and Methods

### 2.1. Materials

#### 2.1.1. Chemicals

Arginine vasopressin (AVP),* SR49059*, neutral red, and Hexamethonium were purchased from Sigma, USA, and chloral hydrate and NaCl were purchased from Tiancheng, Chemical Ltd., China. All chemicals were in technical grade of more than 95% purity. All drugs were dissolved in normal saline (NS) and prepared immediately before use.

#### 2.1.2. Animals

Male adult Wistar rats (a total of 30, 280–320 g body weight, 3 months old) were graciously provided by the Experimental Animal Center of Shandong University, China. Before experiments, all test animals were kept in a temperature-controlled environment on 12-hour light and 12-hour darkness at 22 ± 1°C. Prior to experiments, rats were fasted for 24 hours, and water was provided* ad libitum*. All procedures were conducted in accordance with the National Institutes of Health Guidelines for the Care and Use of Laboratory Animals and were approved by the Institutional Animal Care and Use Committee at Shandong Normal University.

### 2.2. Methods

Rats were selected randomly and divided into five groups, and the experimental design is shown in Table 1.

#### 2.2.1. Recording of Gastric Motility

Animals were anesthetized with an intraperitoneal (i.p.) injection of sodium chloral hydrate (400 mg/kg body mass) and were placed on a small animal operating table in the supine position. A midline laparotomy was performed, and a latex balloon filled with water (5 mm in diameter) was inserted into the pyloric region through a small incision in the forestomach wall. The balloon was connected to a pressure transducer via a thin polyethylene tube. Blood pressure (BP) was monitored via another pressure transducer connected via a cannula inserted into the femoral artery. After the above procedures were completed, the rats were placed on a stereotaxic apparatus; body temperature was kept constant at 37 ± 1°C with a radiant heat lamp. The respiratory frequency (RF) was monitored by a tonotransducer attached to the rat's chest with a clamp. Heart activity was monitored via electrocardiogram (ECG), which was recorded synchronously via the standard leads connected to all four limbs. All the physiological signals were fed into the Biological Function Experiment System (BL-420, Taimeng Technical Co., Chengdu, China). Gastric motility (GM) was recorded for at least 30 min before microinjection and lasted for 30 min after microinjection. The recording of BP, RF, and ECG was made simultaneously with gastric motility.

#### 2.2.2. Stereotaxis and Microinjection

The rats were placed on a stereotaxic apparatus (Stoelting 51600, USA), and the dorsal surface of the medulla was exposed through an occipital craniotomy. A glass micropipette (30–50 *μ*m, external tip diameter) was connected with a microsyringe (volume 1 *μ*L, Anting Scientific Instrument Company, Shanghai, China) via polyethylene tube filled with NS. After removal of the dura, the micropipette tip was stereotaxically introduced into the DMV according to the atlas of Paxinos and Watson (5th edition, coordinates: 0.5–0.9 mm rostral to the obex, a depth of 0.4–0.5 mm below the surface of the obex, 0.5 mm lateral to the midline). The microinjection of AVP into the DMV was performed in a volume of 50 nL for 10–15 sec. In order to investigate the possible central effect of AVP in the DMV on gastric function, a specific V1 receptor antagonist* SR49059* was also administered to rats before each microinjection of AVP. Hexamethonium (autonomic ganglionic cholinergic receptor blockade) was also delivered via intravenous injection before the microinjection of AVP, to explore the possible peripheral mechanisms of AVP on gastric function.

#### 2.2.3. After Treatment

**Figure 1:**
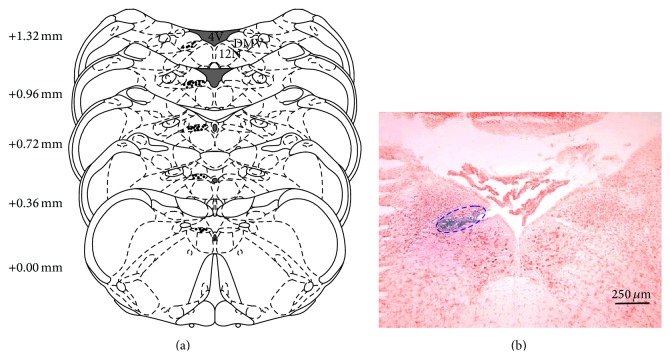
Histological sites of micropipette tips in the DMV of the rat. Reconstruction detailing microinjection sites of chemical into the DMV in the brain atlas, with the dots indicating the micropipette tips. Numbers indicate distance from the obex in the anteroposterior plane. 4V: the 4th ventricle, 12N: the hypoglossal nucleus (a). Representative brain stem section showing the position of DMV, with ellipse and blue macula indicating DMV (b).

At the end of the experiment, 2% potamine sky blue (50 nL) was injected into the same microinjection site. The rats were then deeply anesthetized by intraperitoneal injections of sodium pentobarbital (100 mg/kg body weight) and were perfused transcardially with NS followed by 4% paraformaldehyde. The brains were removed and fixed in 4% paraformaldehyde with 20% sucrose for 2 days. The bulbar region was frozen, serially sectioned (40 *μ*m), and stained with neutral red to facilitate the identification of the micropipette tip in the DMV (Figure 1). All the tip sites of the microinjection were identified, and the data from the rats with the tip sites located outside the DMV were excluded in the analysis. Images of brain sections were taken under identical conditions with a microscope (Olympus Co., Japan) coupled with a digital camera.

### 2.3. Data Analysis

**Figure 2:**
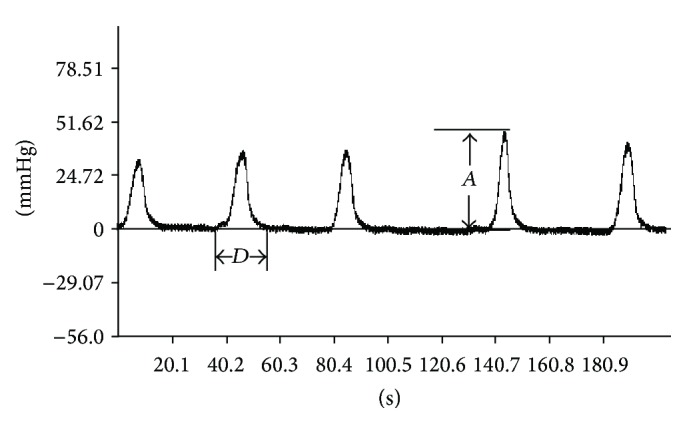
Representative gastric contraction wave in rat, showing the duration (*D*) and amplitude (*A*).

Total duration (the sum of wave durations per 5 min), total amplitude (the sum of wave amplitudes per 5 min), and motility index (MI) of gastric contraction waves within 5 min before microinjection and three consecutive 5 min values after microinjection were measured. In this study, the duration of each contraction wave was defined as the timespan between the onset of ascending phase and the offset of descending phase from the baseline, and the amplitude was defined as the altitude of each contraction wave (Figure 2). The motility index was defined as the product of amplitude and duration of each contraction wave. Inhibitory rates (IR) were applied to estimate the changing degree of gastric motility before and after microinjection; namely, IR  (%) = 100% × (the value before microinjection − the value after microinjection)/the value before microinjection. Changes in BP (mmHg, mean arterial pressure), RF (times/min), and HR (beats/min) were monitored before and after microinjection for 60 sec.

Variables were analyzed using SPSS 13.0 software (SPSS Inc., Chicago, IL, USA) and presented as mean ± SEM. Data were analyzed using one-way analysis of variance followed by Student-Newman-Keuls multiple-comparisons or Student's *t*-test. Statistical significance was set at *P* < 0.05.

## 3. Results

### 3.1. Effects of Different Doses of AVP on Gastric Motility

**Figure 3:**
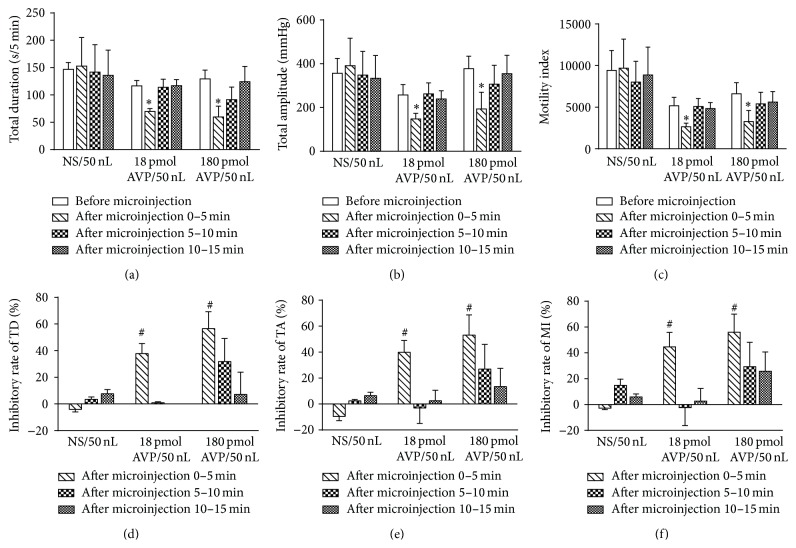
Representative data for gastric motility before and after microinjection of AVP (18 and 180 pmol) or normal saline (NS) into the DMV. Mean data of total duration (a), total amplitude (b), and motility index (c) before microinjection and after microinjection 0–5 min, 5–10 min, and 10–15 min. Average inhibitory rate of total duration (d), total amplitude (e), and motility index (f) in the first 5 min, second 5 min, and third 5 min. ^*∗*^ *P* < 0.05, versus before microinjection; ^#^ *P* < 0.05, versus NS group in the same period.

As shown in Figure 3, the microinjection of AVP (180 pmol or 18 pmol) into the DMV caused a significant inhibition of gastric motility within 5 min (*P* < 0.05) in groups 1 and 2 (Figures 3(a)–3(c)). The inhibitory rates of AVP in group 1 microinjected into the DMV (the first 5 min after microinjection) for total duration, total amplitude, and motility index were 56.50%, 53.15%, and 56.08%, respectively. These percentages were higher than those of AVP in group 2, which suggested that the inhibitory effect of AVP on gastric motility showed a dose-dependent trend in the first 5 min after microinjection (Figures 3(d)–3(f)). In the following 5 minutes in group 1 and group 2, the inhibition of the gastric motility decreased gradually. Ten minutes later after microinjection of AVP, gastric contraction waves returned to normal. No significant inhibition of gastric motility was observed in group 3, in which AVP was substituted for NS (*P* > 0.05).

**Figure 4:**
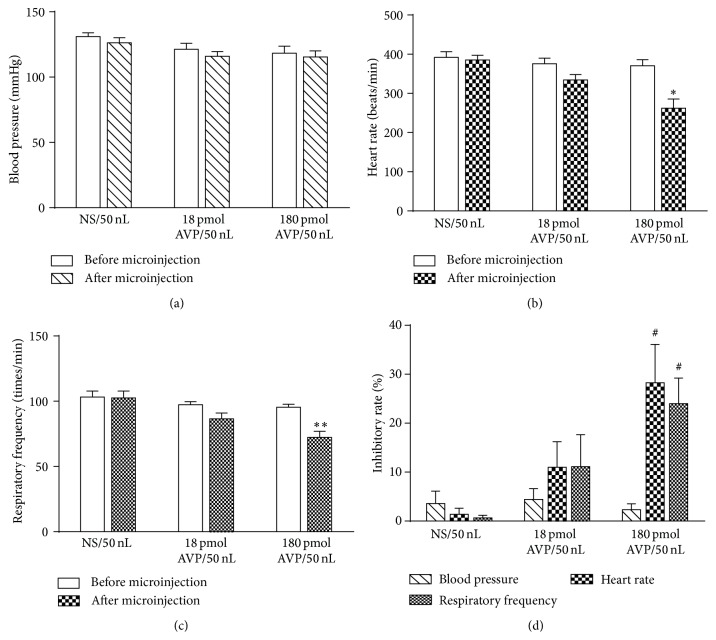
Effects of AVP (18 and 180 pmol) or normal saline (NS) microinjected into the DMV on blood pressure, heart rate, and respiratory frequency. Representative effects of AVP or NS on blood pressure (a) and heart rate (b) and respiratory frequency (c) before and after microinjection. The inhibitory rate of AVP or NS on blood pressure and heart rate and respiratory frequency (d). ^*∗*^ *P* < 0.05 and ^*∗∗*^ *P* < 0.01, versus before microinjection; ^#^ *P* < 0.05, versus NS group in the same period.

As shown in Figure 4, the microinjection of 180 pmol AVP in group 1 significantly decreased the respiratory frequency and the heart rate (after microinjection, *P* < 0.01 or *P* < 0.05). The inhibitory rates of 180 pmol AVP on the respiratory frequency and the heart rate were 23.99% and 28.26%, respectively. However, in group 2 or group 3, the administration of NS or 18 pmol AVP on the respiratory frequency and the heart rate resulted in no significant difference (*P* > 0.05). Blood pressure in three groups was similar.

### 3.2. Effects of AVP on Gastric Motility with Pretreatment of* SR49059* or Hexamethonium

**Figure 5:**
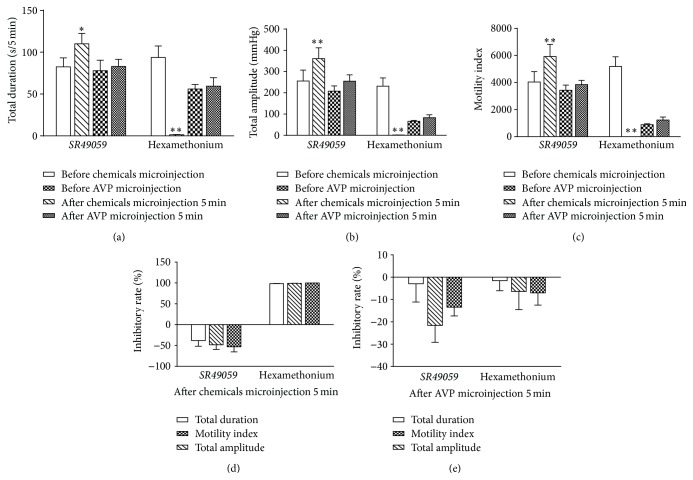
Effects of AVP (180 pmol) on gastric motility with pretreatment of* SR49059* (320 pmol) or Hexamethonium (8 *μ*mol). Mean data of total duration (a) and total amplitude (b) and motility index (c) before microinjection and after microinjection 0–5 min, 5–10 min, and 10–15 min. Average inhibitory rate of total duration, total amplitude, and motility index before and after microinjection of* SR49059* (d) and Hexamethonium (e). ^*∗*^ *P* < 0.05 and ^*∗∗*^ *P* < 0.01, versus before microinjection.

In group 4, 320 pmol* SR49059* was microinjected into the DMV of normal rats, and gastric motility was stimulated within 5 min (*P* < 0.05; *P* < 0.01) (Figures 5(a)–5(c)). The inhibitory rates of SR49059 for total duration, total amplitude, and motility index were −38.28%, −48.47%, and −53.14%, respectively (Figure 5(d)). After 15 min, 180 pmol AVP was delivered into the same site to investigate the effect of AVP on gastric motility in the presence of* SR49059*. The results showed that AVP had no effect on gastric motility in the presence of* SR49059* (Figures 5(a)–5(c) and 5(e)).

In group 5, Hexamethonium was delivered via intravenous injection through the femoral vein in normal rats, and gastric motility was completely inhibited (*P* < 0.01) (Figures 5(a)–5(c)). The inhibitory rates of total duration, total amplitude, and motility index were 98.43%, 99.26%, and 99.96%, respectively (Figure 5(d)). Half an hour later, gastric contraction waves were restored, but at a relatively stable, low amplitude state. 180 pmol AVP was microinjected into the DMV and no change was observed (Figure 5(e)).

## 4. Discussion

### 4.1. Effects of AVP on Gastric Motility

It is well documented that the PVN is a central nucleus for modulating gastric function. Meanwhile a growing body of evidence on the morphology of PVN-DMV projection has shown that the vasopressinergic fibers from PVN project directly to the DMV [7, 9], suggesting a close relationship between AVP in the DMV and gastric function. In this study, in group 1 or group 2, the microinjection of AVP (180 pmol or 18 pmol) into the DMV produced an inhibition on gastric motility in a dose-dependent manner within 5 min, suggesting that extrinsic AVP participates in modulating the gastric motility. In group 4,* SR49059*, a specific V1 receptor antagonist, injected into the DMV of normal rats significantly increased gastric motility within 5 min, suggesting the involvement of intrinsic AVP from PVN in modulating the gastric motility. In group 2, 18 pmol AVP inhibited the gastric motility but had no effect on the respiratory frequency, the heart rate, and blood pressure, suggesting that the effect of AVP on gastric motility was specific and not due to a global depression linked to systemic action. All findings revealed that AVP participated in the process of modulating gastric motility by activating neurons of DMV, not by systemic action.

Growing evidence indicates the involvement of OT exciting gastric-related neurons of the DMV in modulating the gastric function [17–21]. Despite much research in OT from a clinical and experimental perspective, little is known about whether AVP in the DMV are involved in the process of modulating gastric motility* in vivo* in rats. To our knowledge, there has been no such study so far. AVP and OT are the highly related nonapeptides in structure released by PVN and SON, showing only a little difference in two of all the amino acids. AVP and OT activate the neurons via G-protein coupled receptor and thus could combine with their receptors reciprocally [14]. Our present data strongly demonstrate that the vasopressinergic projection from the PVN to the DMV probably plays a similar role in the gastric functions and produces an excitatory effect on the neurons of DMV as the oxytocinergic system does.

The DMV contains cell bodies of the vagal preganglionic neurons that could control gastric motility, and the vast majority (>95%) of neurons innervating the stomach are cholinergic [22]. Microinjection of L-Glutamate into the rostral or caudal DMV could produce gastric contraction or relaxation, respectively, suggesting a characteristic spatial organization of vagal preganglionic neurons in the DMV [16, 23, 24]. Cruz et al. reported that the vagal efferent pathway which could excite gastric motility was originated from the area of the DMV rostral to calamus scriptorius (CS) and which could inhibit gastric motility was originated from the area of the DMV caudal to CS [25]. However, in the present study, the microinjection sites were distributed in the rostral, middle, and caudal DMV regions. The microinjection of AVP into the whole DMV led to the inhibition of gastric motility. According to the previous results [11], there are AVP receptors in the soma and/or dendrite membranes of neurons in the whole DMV. Therefore, these findings suggest that the AVP-sensitive neurons are wide spread all over the DMV.

### 4.2. Possible Mechanism of the Effect of AVP on Gastric Motility

The effect of AVP injected into the DMV on gastric motility was blocked by* SR49059*. This result was similar to previous findings* in vitro* [26], which reported that AVP (10^−6^ M) could increase the firing rate of a portion of neurons in the DMV and that the excitatory responses could be blocked completely by the selective V1 receptor antagonist. This result strongly suggests that AVP may inhibit the gastric motility by activating the specific AVP receptor in the DMV.

It has been known that the vagal preganglionic fibers originated from the DMV regulate a large component of gastric motility patterns via the enteric nervous system (ENS) and/or the network of pacemaker interstitial Cajal cells (ICC) within the muscle wall of the stomach. In the present study, the gastric contractions were completely inhibited by Hexamethonium (autonomic ganglionic cholinergic receptor blockade), which means that the AVP-sensitive neurons controlling gastric motility should be cholinergic. Thirty minutes later, the gastric contraction waves were restored but at a lower amplitude and no effect of AVP microinjected into the MDV on gastric motility was recorded by pretreatment with Hexamethonium. We hypothesize that a part of the vagal preganglionic fiber endings might establish synaptic contact with ICC and in turn control the activity of gastric smooth muscles under normal physiological state [27]. Hexamethonium blocked the signal transmission within the synapse, and the excitability of ICC was decreased temporarily by the sudden deficiency in controlling of vagal preganglionic fibers but gradually renewed after about 30 minutes. Thus, the restored waves with lower amplitude were largely mediated by nonneural mechanism, namely, were due to slow wave depolarization generated by pacemaker ICC.

Previous studies have demonstrated that the inhibitory pathways of vagal control of gastric function are composed of two types of preganglionic vagal neurons in the DMV. The first type consists of preganglionic cholinergic neurons, synapsed with postganglionic NANC neurons [23, 25, 28, 29], for example, nitrergic/VIPergic neurons in the gastric wall [30]. The second type is preganglionic nitrergic neurons, synapsed with postganglionic NANC neurons [16, 31]. Considering these data, it would be reasonable to assume that AVP plays a role in regulating gastric motility through the preganglionic cholinergic and postganglionic NANC neural pathway, rather than the preganglionic nitrergic and postganglionic NANC neural pathway, but more studies are needed to elucidate the specific types of postganglionic fibers.

### 4.3. Effects of AVP on Respiratory Frequency, Heart Rate, and Blood Pressure

It is also well known that the DMV contains the vagal parasympathetic preganglionic neurons innervating the heart [32, 33] and that the microinjection of OT into the DMV produces a consistent reduction in heart rate [20]. Similarly, in our study, the microinjection of 180 pmol AVP into the DMV significantly inhibited the heart rate (*P* < 0.05). It has been also reported that transfusions of AVP could lead to an increase in airway resistance and a decrease in lung compliance and that AVP microinjected into the areas of the ventrolateral medulla (VLM) could inhibit the phrenic nerve activity and respiration in rat [34]. These results indicate that AVP might cause an inhibitory effect on respiration via both peripheral and central mechanisms. In our study, the effect of AVP injected into DMV on the respiratory frequency was consistent with these previous findings.

AVP is known to have hypertensive effects following intravenous infusion, and the changes of arterial blood pressure frequently alter the intragastric pressure accompanied by the gastric wall blood flow [35]. In this study, blood pressure remained almost constant during the microinjection of different doses of AVP into DMV. All these results suggest that the dosage of AVP is enough to activate the vagal preganglionic neurons in the DMV, and AVP-induced changes in gastric motility are likely attributed to central mechanism, rather than its hypertensive effects.

## Figures and Tables

**Table 1:** The design of the experiment.

Group	Numbers	Chemicals	Dosage or concentration	Volume	Sites of injection	Detecting index
Group 1	6	AVP	180 pmol	50 nL	DMV	GM, BP, HR, and RF
Group 2	6	AVP	18 pmol	50 nL	DMV	GM, BP, HR, and RF
Group 3	6	NS	0.9%	50 nL	DMV	GM, BP, HR, and RF
Group 4	6	*SR49059*	320 pmol	50 nL	DMV	GM
		AVP	180 pmol			
Group 5	6	Hexamethonium	8 *μ*mol	1 mL	Femoral Vein	GM
		AVP	180 pmol	50 nL	DMV	

GM, gastric motility; BP, blood pressure; HR, heart rate; RF, respiratory frequency; NS, normal saline.
